# Supplementary material for: Orally Administered 6:2 Chlorinated Polyfluorinated Ether Sulfonate (F-53B) Causes Thyroid Dysfunction in Rats
Source: Toxics. 2020 Aug 8;8(3):54. doi: 10.3390/toxics8030054 (PMC7560397; doi:10.3390/toxics8030054)
Supplement: Supplementary file 1 [file toxics-08-00054-s001.pdf]

Supplementary Materials: Orally Administered 6:2 Chlorinated Polyfluorinated Ether Sulfonate (F-53B) Causes Thyroid Dysfunction in Rats

So-Hye Hong, Seung Hee Lee, Jun-Young Yang, Jin Hee Lee, Ki Kyung Jung, Ji Hyun Seok, Sung-Hee Kim, Ki Taek Nam, Jayoung Jeong, Jong Kwon Lee and Jae-Ho Oh

Table S1. Hematology data for male rats in the 28-day gavage study of F-53B.

Parameter	Groups (mg/kg/day)			
	**Vehicle Control**	**5**	**20**	**100**
RBCs (× 10^6 cells/μL)	8.65±0.491	8.99±0.322	8.92±0.264	8.74±0.245
HGB (g/dL)	16.4±0.67	16.7±0.63	17.0±0.28	16.9±0.40
HCT (%)	46.9±2.72	48.8±1.86	49.1±1.47	48.5±1.07
MCV (fL)	54.2±0.84	54.3±1.53	55.0±1.14	55.4±0.50
MCH (pg)	19.0±0.91	18.5±0.87	19.0±0.41	19.3±0.63
MCHC (g/dL)	35.0±1.52	34.2±1.26	34.6±0.82	34.8±1.05
PLTs (× 10^3 cells/μL)	986±110.1	980±116.5	984±72.7	991±72.9
WBCs (× 10^3 cells/μL)	8.89±2.146	8.94±2.353	8.46±2.191	7.56±1.199
NEU (% of WBCs)	15.3±3.56	15.8±5.23	13.6±2.98	16.7±9.74
LYM (% of WBCs)	78.8±4.29	78.3±6.14	80.3±2.78	78.0±9.33
MONO (% of WBCs)	3.3±1.28	3.8±1.46	3.5±0.49	3.2±0.94
EOS (% of WBCs)	1.4±1.08	0.7±0.25	1.2±0.30	1.2±0.35
BASO (% of WBCs)	0.2±0.07	0.2±0.08	0.3±0.08	0.2±0.07
LUC (% of WBCs)	0.9±0.55	1.2±0.42	1.1±0.41	0.9±0.41
RET (%)	2.0±0.198	1.86±0.324	2.19±0.289	1.94±0.180

RBC, red blood cell; HGB, hemoglobin; HCT, hematocrit; MCV, mean corpuscular volume; MCH, mean corpuscular hemoglobin; MCHC, mean corpuscular hemoglobin concentration; PLT, platelet; WBC, white blood cell; NEU, neutrophil; LYM, lymphocyte; MONO, monocyte; EOS, eosinophil; BASO, basophil; LUC, large unstained cell; RET, reticulocyte. Data are expressed as mean ± SD values (n = 7/group). *Significantly different from the vehicle control group ($p < 0.05$) Statistics: One-way analysis of variance (ANOVA) followed by the Dunnett's test.

Table S2. Hematology data for female rats in the 28-day gavage study of F-53B s.

Parameter	Groups (mg/kg/day)			
	**Vehicle Control**	**5**	**20**	**100**
RBCs (× 10^6 cells/μL)	7.93±0.319	7.97±0.329	7.9±0.22	8.08±0.344
HGB (g/dL)	14.9±0.37	15.1±0.32	15.1±0.36	15.8±0.51
HCT (%)	43.6±0.79	43.3±1.46	43.4±1.09	44.7±1.95
MCV (fL)	43.6±2.94	54.4±0.92*	55.1±1.05*	55.4±1.34*
MCH (pg)	18.8±0.42	19.0±0.68	19.2±0.41	19.5±0.63
MCHC (g/dL)	34.3±0.73	35.0±0.96	34.8±0.75	35.3±0.71
PLTs (× 10^3 cells/μL)	1103±78.1	1111±65.9	1124±56.1	1175±81.1
WBCs (× 10^3 cells/μL)	5.78±1.247	5.81±1.652	5.34±0.545	5.23±1.658
NEU (% of WBCs)	15.3±5.26	15.8±9.99	16.6±5.71	13.5±3.78
LYM (% of WBCs)	79.1±5.67	78.9±10.39	77.7±5.85	81.0±3.81
MONO (% of WBCs)	3.3±0.72	2.9±0.74	3.0±0.62	2.7±0.92
EOS (% of WBCs)	0.9±0.29	1.1±0.48	1.5±0.46	1.2±0.49
BASO (% of WBCs)	0.2±0.08	0.2±0.04	0.2±0.10	0.1±0.10
LUC (% of WBCs)	1.2±0.17	1.1±0.46	1.0±0.33	1.0±0.53
RET (%)	2.6±0.69	2.4±0.46	2.5±0.31	2.9±0.61

RBC, red blood cell; HGB, hemoglobin; HCT, hematocrit; MCV, mean corpuscular volume; MCH, mean corpuscular hemoglobin; MCHC, mean corpuscular hemoglobin concentration; PLT, platelet; WBC, white blood cell; NEU, neutrophil; LYM, lymphocyte; MONO, monocyte; EOS, eosinophil; BASO, basophil; LUC, large unstained cell; RET, reticulocyte. Data are expressed as mean ± SD values (n = 7/group). *Significantly different from the vehicle control group ($p < 0.05$) Statistics: One-way analysis of variance (ANOVA) followed by the Dunnett's test.

Table S3. Clinical biochemistry data for male rats in the 28-day gavage study of F-53B.

Parameter	Groups (mg/kg/day)			
	**Vehicle Control**	**5**	**20**	**100**
ALT (IU/L)	43.19±7.730	45.06±9.208	54.60±29.974	39.56±6.988
AST (IU/L)	125.23±23.005	125.63±20.611	131.27±29.366	119.03±25.106
ALP (IU/L)	340.47±80.153	339.63±60.051	335.96±86.256	305.66±40.462
GGT (IU/L)	0.51±0.240	0.31±0.108	0.77±0.629	0.35±0.140
BUN (mg/dL)	21.44±2.946	21.77±2.578	21.23±1.746	20.29±1.079
CREA (mg/dL)	0.64±0.072	0.69±0.116	0.67±0.073	0.65±0.052
TP (g/dL)	5.96±0.175	6.00±0.234	6.03±0.066	5.97±0.110
T-BIL (mg/dL)	0.18±0.036	0.19±0.039	0.20±0.016	0.19±0.016
GLU (mg/dL)	126.89±17.108	129.79±13.362	128.21±19.569	136.64±24.992
TG (mg/dL)	33.90±8.909	28.20±6.383	30.81±7.936	38.26±9.567
ALB (g/dL)	3.88±0.037	3.88±0.140	3.91±0.057	3.93±0.034
A/G ratio	1.87±0.114	1.83±0.098	1.84±0.056	1.93±0.056
Ca (mg/dL)	9.86±0.294	9.96±0.450	9.82±0.309	9.80±0.350
Na (mmol/L)	141.57±1.134	142.57±2.299	142.71±0.951	143.29±1.113
Cl (mmol/L)	101.71±0.488	102.86±0.690*	103.29±1.254*	104.14±0.690*
K (mmol/L)	4.87±0.233	4.93±0.337	4.86±0.264	4.91±0.191
IP (mg/dL)	8.72±0.458	8.90±0.978	8.39±1.123	8.29±0.763

ALT, alanine aminotransferase; AST, aspartate aminotransferase; ALP, alkaline phosphatase; GGT, gamma-glutamyl transferase; BUN, blood urea nitrogen; CREA, creatinine; TP, total protein; T-BIL, total bilirubin; T-CHOL, cholesterol; GLU, glucose; TG, triglyceride; ALB, albumin; A/G ratio, albumin/globulin ratio; Ca, calcium; Na, sodium; Cl, chloride; K, potassium; IP, phosphorus; HDL, high-density lipoprotein; LDL, low-density lipoprotein. Data are expressed as mean ± SD values (n = 7/group). *Significantly different from the vehicle control group ($p < 0.05$). Statistics: One-way analysis of variance (ANOVA) followed by the Dunnett's test.

Table S4. Clinical biochemistry data for male rats in the 28-day gavage study of F-53B.

Parameter	Groups (mg/kg/day)			
	**Vehicle Control**	**5**	**20**	**100**
ALT (IU/L)	35.20±3.097	34.83±6.698	37.99±10.808	36.70±3.462
AST (IU/L)	110.50±27.220	104.29±21.954	111.00±20.353	112.10±17.523
ALP (IU/L)	249.77±31.380	250.94±41.536	242.17±41.349	237.51±37.243
GGT (IU/L)	0.84±0.234	0.68±0.324	0.84±0.253	0.90±0.283
BUN (mg/dL)	19.21±1.642	22.53±4.673	19.34±4.451	21.86±4.275
CREA (mg/dL)	0.68±0.073	0.74±0.062	0.72±0.114	0.76±0.083
TP (g/dL)	6.25±0.221	6.24±0.226	6.30±0.341	6.61±0.242*
T-BIL (mg/dL)	0.24±0.053	0.24±0.027	0.22±0.019	0.25±0.046
GLU (mg/dL)	111.77±15.741	109.34±16.450	116.90±3.804	113.71±19.619
TG (mg/dL)	21.44±6.702	24.73±7.372	22.93±2.453	21.64±10.168
ALB (g/dL)	4.06±0.109	4.13±0.156	4.14±0.184	4.33±0.165
A/G ratio	1.86±0.069	1.96±0.079	1.92±0.148	1.90±0.076
Ca (mg/dL)	10.05±0.228	10.06±0.245	9.97±0.113	10.14±0.228
Na (mmol/L)	142.71±0.756	143.57±0.535	144.00±1.414	145.00±1.633*
Cl (mmol/L)	105.29±1.380	106.14±1.215	107.29±0.756*	107.29±2.138*
K (mmol/L)	4.42±0.138	4.42±0.289	4.60±0.319	4.76±0.486
IP (mg/dL)	8.47±0.795	8.37±0.748	7.85±0.871	8.16±1.420

ALT, alanine aminotransferase; AST, aspartate aminotransferase; ALP, alkaline phosphatase; GGT, gamma-glutamyl transferase; BUN, blood urea nitrogen; CREA, creatinine; TP, total protein; T-BIL, total bilirubin; T-CHOL, cholesterol; GLU, glucose; TG, triglyceride; ALB, albumin; A/G ratio, albumin/globulin ratio; Ca, calcium; Na, sodium; Cl, chloride; K, potassium; IP, phosphorus; HDL, high-density lipoprotein; LDL, low-density lipoprotein. Data are expressed as mean ± SD values (n = 7/group). *Significantly different from the vehicle control group ($p < 0.05$). Statistics: One-way analysis of variance (ANOVA) followed by the Dunnett's test.

Table S5. Relative organ weight data for male rats in the 28-day gavage study of F-53B.

Parameter	Groups (mg/kg/day)			
	**Vehicle Control**	**5**	**20**	**100**
Body weight (g)	388.13±21.907	363.58±16.671	370.66±17.582	373.15±21.014
Liver (%)	2.554±0.0717	2.500±0.0943	2.500±0.0930	2.581±0.0682
Kidneys (%)	0.647±0.0199	0.653±0.0285	0.664±0.0209	0.647±0.0309
Adrenal glands (%)	0.014±0.0018	1.015±0.0017	0.014±0.0015	0.014±0.0008
Heart (%)	0.344±0.0211	0.353±0.0213	0.343±0.0219	0.346±0.0205
Brain (%)	0.517±0.0282	0.534±0.0215	0.535±0.0323	0.530±0.0253
Pituitary gland (%)	0.003±0.0002	0.003±0.0005	0.003±0.0007	0.003±0.0009
Spleen (%)	0.214±0.0155	0.206±0.0174	0.214±0.0255	0.206±0.0155
Thymus (%)	0.120±0.0140	0.099±0.0187*	0.112±0.0144	0.096±0.0106*
Testes (%)	1.004±0.0734	1.143±0.0790*	1.072±0.0315	1.073±0.0535
Epididymis (%)	0.338±0.0290	0.362±0.0309	0.366±0.0221	0.359±0.0246
Prostate, seminal vesicle and coagulating gland (%)	0.689±0.0504	0.698±0.0750	0.680±0.0884	0.695±0.0646

Data are expressed as mean ± SD values (n = 7/group). *Significantly different from the vehicle control group ($p < 0.05$) Statistics: One-way analysis of variance (ANOVA) followed by the Dunnett's test.

Table S6. Relative organ weight data for male rats in the 28-day gavage study of F-53B.

Parameter	Groups (mg/kg/day)			
	**Vehicle Control**	**5**	**20**	**100**
Body weight (g)	219.79±6.615	223.45±15.666	214.96±6.592	209.93±6.639
Liver (%)	2.525±0.0558	2.471±0.0822	2.552±0.1260	2.493±0.1338
Kidneys (%)	0.683±0.0361	0.650±0.0283	0.643±0.0310	0.654±0.0255
Adrenal glands (%)	0.030±0.0017	0.031±0.0022	0.032±0.0020	0.032±0.0035
Heart (%)	0.379±0.0156	0.375±0.0246	0.394±0.0179	0.380±0.0138
Brain (%)	0.816±0.0379	0.798±0.0406	0.827±0.0268	0.850±0.0319
Pituitary gland (%)	0.006±0.0006	0.005±0.0014	0.006±0.0006	0.006±0.0003
Spleen (%)	0.270±0.0290	0.266±0.0225	0.266±0.0469	0.272±0.0226
Thymus (%)	0.144±0.0159	0.146±0.0117	0.154±0.0239	0.272±0.0226
Ovaries (%)	0.040±0.0030	0.039±0.0046	0.040±0.0062	0.039±0.0071
Uterus (%)	0.227±0.0380	0.250±0.1359	0.294±0.1291	0.299±0.1188

Data are expressed as mean ± SD values (n = 7/group). *Significantly different from the vehicle control group ($p < 0.05$) Statistics: One-way analysis of variance (ANOVA) followed by the Dunnett's test.
